# Cinnamon Bark for Tooth-Ache

**Published:** 1884-11

**Authors:** 


### Cinnamon Bark for Tooth-Ache.

In the *North Carolina Medical Journal*, April, 1884, Dr. J. R. Irwin writes that one of the best and most pleasant things that can be used to relieve this painful state of the dental nerves, is chewing cinnamon bark. It destroys the sensibility of the nerves and suspends the pain immediately, if the bark is of good quality. After repeated trials, and in different cases, he is convinced that it is generally as efficacious as any of the other remedies suggested for odontalgia, and not attended with the unpleasant consequences of creasote, carbolic acid, etc., which relieve the pain, but leave the mouth as sore and painful as the tooth was previously, though these results are usually due to carelessness in using.

